# Characterization of SARS‐CoV‐2 and common cold coronavirus‐specific T‐cell responses in MIS‐C and Kawasaki disease children

**DOI:** 10.1002/eji.202149556

**Published:** 2021-10-27

**Authors:** Li‐En Hsieh, Alba Grifoni, John Sidney, Chisato Shimizu, Hiroko Shike, Nanda Ramchandar, Elizabeth Moreno, Adriana H. Tremoulet, Jane C. Burns, Alessandra Franco

**Affiliations:** ^1^ Department of Pediatrics School of Medicine University of California, San Diego La Jolla CA USA; ^2^ Division of Vaccine Discovery La Jolla Institute for Immunology La Jolla CA USA; ^3^ Department of Pathology and Laboratory Medicine Penn State Milton S. Hershey Medical Center Hershey PA USA

**Keywords:** Kawasaki disease, multisystem inflammatory syndrome in children (MIS‐C), SARS‐CoV‐2, T cells, T‐cell memory

## Abstract

The immunopathogenesis of multisystem inflammatory syndrome (MIS‐C) in children that may follow exposure to SARS‐CoV‐2 is incompletely understood. Here, we studied SARS‐CoV‐2‐specific T cells in MIS‐C, Kawasaki disease (KD), and SARS‐CoV‐2 convalescent controls using peptide pools derived from SARS‐CoV‐2 spike or nonspike proteins, and common cold coronaviruses (CCC). Coordinated CD4+ and CD8+ SARS‐CoV‐2‐specific T cells were detected in five MIS‐C subjects with cross‐reactivity to CCC. CD4+ and CD8+ T‐cell responses alone were documented in three and one subjects, respectively. T‐cell specificities in MIS‐C did not correlate with disease severity and were similar to SARS‐CoV‐2 convalescent controls. T‐cell memory and cross‐reactivity to CCC in MIS‐C and SARS‐CoV‐2 convalescent controls were also similar. The chemokine receptor CCR6, but not CCR9, was highly expressed on SARS‐CoV‐2‐specific CD4+ but not on CD8+ T cells. Only two of 10 KD subjects showed a T‐cell response to CCC. Enumeration of myeloid APCs revealed low cell precursors in MIS‐C subjects compared to KD. In summary, children with MIS‐C mount a normal T‐cell response to SARS‐CoV‐2 with no apparent relationship to antecedent CCC exposure. Low numbers of tolerogenic myeloid DCs may impair their anti‐inflammatory response.

## Graphical abstract

T cells derived from children with multisystem inflammatory syndrome (MIS‐C) respond to SARS‐CoV‐2 spike and nonspike peptide megapools. SARS‐CoV‐2‐specific memory T cells and homing receptors showed different patterns between CD4+ and CD8+ T cells. MIS‐C subjects had very low myeloid dendritic cells in circulation including ILT‐4+ CD4+ tolerogenic dendritic cells.

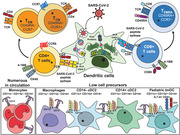

## Introduction

A unique feature of the SARS‐CoV‐2 pandemic was the low numbers of children suffering serious illness when acutely infected with SARS‐CoV‐2. However, in mid‐March 2020, pediatricians in communities in Western Europe, the UK, and the eastern US noted an increased number of children presenting with fever and evidence of severe systemic inflammation requiring admission to an intensive care unit. The majority of children had evidence of exposure to SARS‐CoV‐2 with detectable antibody to the virus [1]. A hallmark of these cases was heart failure leading to shock, and the absence of significant pulmonary disease, but with clear signs of hyperinflammation. Children presenting with high fever, multiorgan dysfunction, and antecedent exposure to the coronavirus were defined as “*Multisystem inflammatory syndrome‐in children (MIS‐C)”* [2, 3]. The clinical presentation in these patients shared features with Kawasaki disease (KD), an acute pediatric vasculitis that affects the coronary arteries. Although the two diseases are distinct, they share an acute inflammatory response [4]. Thus, KD provides a pediatric disease model to compare and contrast with the immune repertoire in MIS‐C.

T cells play an important role in controlling SARS‐CoV‐2 infection. The patterns of immunodominance of different SARS‐CoV‐2 antigens measured by epitope‐specific T‐cell responses in adult convalescent SARS‐CoV‐2 patients and unexposed subjects have been described [5, 6]. In those studies, the SARS‐CoV‐2 proteome was probed using 1925 peptides (9 to 15 amino acids in length) spanning the whole genome, allowing for detection of HLA class II‐restricted CD4+ T‐cell responses and HLA class I‐restricted CD8+ T‐cell responses [5, 6]. Coordinated CD4+ Th and CD8+ cytotoxic T cell (CTL) responses were associated with reduced disease severity indicating a clear role for early T‐cell responses, in concert with antibodies, in the protective immunity to SARS‐CoV‐2 [7, 8]. It was hypothesized that previous exposure to common cold coronaviruses (CCC) might correlate with a less severe clinical outcome in SARS‐CoV‐2 infection [9, 10].

Here, we studied SARS‐CoV‐2‐specific T‐cell responses in children with MIS‐C to determine their ability to recognize SARS‐CoV‐2 epitopes and to examine the extent to which the magnitude of the SARS‐CoV‐2 responses and cross‐reactivity with CCC epitopes might shape their clinical response. SARS‐CoV‐2‐specific T‐cell responses in MIS‐C were compared to convalescent SARS‐CoV‐2‐infected controls. We also determined the T‐cell memory phenotype and the expression of CCR6 on SARS‐CoV‐2‐specific T cells. We included in the study the CD4+ and CD8+ T‐cell responses to CCC and characterized the APC lineages in children with MIS‐C and in KD subjects whose T cells were harvested both before and during the SARS‐CoV‐2 pandemic.

## Results

### Detection of SARS‐CoV‐2 CD4+ and CD8+ T‐cell responses in MIS‐C subjects

**Figure 1:**
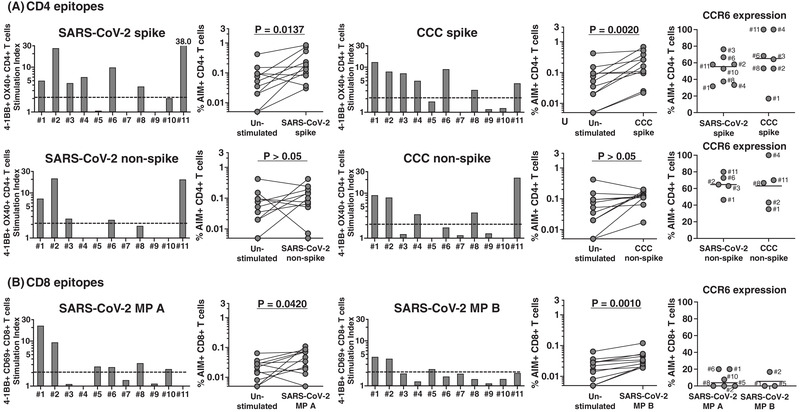
**CD4+ and CD8+ T‐cell responses to peptide megapools derived from SARS‐CoV‐2 and CCC and their CCR6 expression in MIS‐C subjects**. PBMC from 11 MIS‐C subjects were separated from heparinized whole blood using Histopaque and stimulated with peptide megapools derived from SARS‐CoV‐2 CD4 spike (253 epitopes), CCC CD4 spike (124 epitopes), SARS‐CoV‐2 CD4 nonspike (221 epitopes), CCC CD4 nonspike (129 epitopes), or SARS‐CoV‐2 CD8 (MP A and B, 314 epitopes each). Twenty‐four hours after stimulation, cell preparations were collected and stained by monoclonal antibodies to study the T‐cell activation in response to megapool stimulation and the CCR6 expression of the antigen‐specific CD4+ and CD8+ T cells by flow cytometry. The T‐cell response to the megapools is shown as stimulation index (SI) by calculating the percentage of activated T cells in response to peptide megapools divided by the percentage of activated T cells in the unstimulated controls. A stimulation index (SI) ≥2 was considered a positive T‐cell response to the megapool. **(A)** CD4+ T‐cell responses and CCR6 expression to CD4 peptide megapools in individual MIS‐C subjects. CD4+ T‐cell activation was defined with the double expression of 4‐1BB+ OX40+ in gated CD4+ T cells (AIM+). Seven (#1, 2, 3, 4, 6, 8, and 11) of the 11 subjects showed CD4+ T‐cell responses to SARS‐CoV‐2 and CCC CD4 spike megapools. Three subjects (#1, 2, and 11) also showed CD4+ T‐cell responses to both SARS‐CoV‐2 and CCC CD4 nonspike megapools. CD4+ T‐cell response toward peptide antigens derived from spike protein of both SARS‐CoV‐2 (*p* = 0.0137) and CCC (*p* = 0.002) was greater than the response to peptide antigens derived from nonspike protein. The percentage of CCR6+ CD4+ T cells was high in the antigen‐specific T‐cell populations. **(B)** CD8+ T‐cell responses and CCR6 expression to CD8 peptide megapools in each individual MIS‐C subject. CD8+ T‐cell activation was measured as 4‐1BB+ CD69+ (AIM+) following megapools stimulation. Six subjects (#1, 2, 5, 6, 8, and 10) responded to SARS‐CoV‐2 MP A and three (#1, 2, and 5) also responded to SARS‐CoV‐2 MP B. The percentage of CCR6+ CD8+ T cells was low in the antigen‐specific T‐cell populations. Symbols represent the data derived from each individual subject. The study of 11 subjects was completed in seven independent experiments (one or two subjects/experiment depending upon enrollment). Comparisons of the percentage of AIM+ T cells in the unstimulated control and peptide megapool‐stimulated cultures were tested by Wilcoxon signed rank test.

We enrolled 11 MIS‐C subjects (Table 1) to study the T‐cell response to spike and nonspike SARS‐CoV‐2 peptide pools. PBMC were stimulated in vitro with different peptide megapools tailored to capture Th CD4+ T‐cell responses and CTL responses. CD4+ T‐cell responses were evaluated by the activation‐induced cell markers (AIM) assay by measuring the expression of two costimulatory molecules, OX‐40 and 4‐1BB, 24 h after incubation of PBMC cultures with peptide megapools. CD8+ T‐cell responses were evaluated by measuring the expression of 4‐1BB and CD69, 24 h after incubation of PBMC cultures with peptide megapools (Fig. 1 and Supporting information Figs. S1A and S2).

**Table 1:** MIS‐C subjects enrolled in this study

													SARS‐CoV‐2	

Subjects	Age (yrs)	Sex	Illness dayat phlebotomy	*Z*max^a^	Worst LVEF^b^	WBC^c^ (103/μL)	Absolute lymphocyte count	CRP (mg/dL)	ESR (mm/h)	d‐dimer (μg/mL)	Ferritin (ng/mL)	BNP Max^d^ (pg/mL)	PCR	Ab
1	6.0	M	15	3.2	60	4.8	816	19.8	22	2.16	323	10	−	+
2	9.7	M	18	2.1	63	4.7	376	23.8	44	1.31	960	612	−	+
3	8.1	F	20	1.3	70	4.7	1833	0.8	17	1.96	1179	10	−	−
4	7.8	M	20	1.2	72	5.1	1020	21.3	48	5.27	223	241	−	+
5	5.6	F	21	2.0	57	11.6	1392	18.1	83	1.31	140	10	−	−
6	10.5	M	22	1.8	31	13.3	798	31.3	59	2.56	1019	1349	−	+
7	0.8	F	24	1.6	65	16	4160	15.9	3	20	82	10	−	−
8	10.5	M	24	0.9	47	4.9	147	26.8	65	12.77	1441	2590	−	+
9	4.8	M	32	2.5	51	10.1	707	15.7	38	3.02	460	847	−	+
10	11.3	M	42	1.2	58	11.3	1017	19.6	36	1.71	225	561	−	+
11	16.0	F	52	3.5	56	10.9	2289	20.7	90	2.34	1080	30	−	+
12	10.4	M	21	1.3	64	10.5	315	7.1	21	4.61	241	296	−	+
13	8.2	F	25	1.2	57	11.3	1130	19.3	74	1.97	570	245	−	+
14	7.5	M	29	1.9	56	7.2	360	26.3	55	4.71	413	471	−	+
15	9.1	F	20	0.7	49	17.7	2124	28.4	90	3.46	1076	227	−	+
16	3.7	F	21	1.3	63	8.4	2133.6	8.1	17	1.79	204	92	−	+
17	12.9	M	18	2.7	54	11.1	111	29.4	69	5.11	910	717	−	+
18	12.6	M	23	3.6	47	8.6	0	16.9	16	4.38	166	226	−	+
19	7.2	M	54	1.7	66	14.3	1001	5.4	20	2.07	283	25	−	+
20	6.6	M	19	1.2	48	NA	NA	20.0	47	11.00	180	620	−	+

^a^ *Z*max: Maximum *Z* score (internal diameter normalized for body surface area) for the right and left anterior descending coronary arteries.

^b^ Worst LVEF: Lowest LVEF measurement during hospitalization.

^c^ Laboratory data are pretreatment except for BNP.

^d^ BNPmax: Maximum BNP measurement during hospitalization.

*Abbreviations*: BNP, brain natriuretic peptide; CRP, C‐reactive protein; ESR, erythrocyte sedimentation rate; LVEF, left ventricular ejection fraction; WBC, white blood cell count; PCR, polymerase chain reaction; Ab, antibodies; NA, not available.

Nine of the 11 MIS‐C subjects responded to SARS‐CoV‐2 megapools. The two nonresponders were the youngest children in the cohort (Table 1, #7, 8 months and #9, 4.8 years of age), who had no detectable T‐cell response to SARS‐CoV‐2 or to CCC (Fig. 1). HLA alleles of these two subjects, (#7 and #9) were shared by other individuals in the cohort who did respond to SARS‐CoV‐2 peptides (Table 2).

**Table 2:** HLA types of 11 MIS‐C subjects

#	HLA‐A	HLA‐B	HLA‐C	HLA‐DRB1	HLA‐DRB3	HLA‐DRB4	HLA‐DRB5	HLA‐DQA1	HLA‐DQB1	HLA‐DPA1	HLA‐DPB1
1	*30:02P *34:02P	*44:03P *53:01P	*04:01P *04:01P	*07:01P *15:03P	−	*01:01P	*01:01P	*01:02P *02:01P	*02:01P *06:02P	*02:01P *03:01P	*04:02P *17:01P
2	*23:01P *24:07P	*35:05 *45:01	*04:01P *06:02P	*11:01P *12:02P	*02:02P *03:01P	−	−	*01:02P *06:01P	*03:01P *06:02P	*01:03P *02:01P	*13:01P *18:01P
3	*02:01P *03:01P	*35:01P *35:03P	*04:01P *04:01P	*04:03P *11:04P	*02:02P	01:01P	−	*03:01P *05:01P	*03:01P *03:02P	*01:03P *01:03P	*02:01P *04:01P
4	*24:02P *68:01P	*39:06P *40:08	*03:04P *07:02P	*04:04P *14:06P	*01:01P	*01:01P	−	*03:01P *05:01P	*03:01P *03:02P	*01:03P *01:03P	*04:02P *04:02P
5	*01:01P *30:01P	*08:01P *58:01P	*07:01P *07:01P	*01:02P *04:01P	−	*01:01P	−	*01:01P *03:01P	*03:02P *05:01P	*01:03P *02:01P	*04:01P *19:01P
6	*30:01 *32:01	*13:02P *27:02P	*02:02P *06:02P	*04:02P *04:05P	−	*01:01P *01:01P	−	*03:01P *03:01P	*03:02P *03:02P	*01:03P *02:01P	*01:01P *04:02P
7	*24:07P *24:07P	*38:02P *38:02P	*07:02P *07:02P	*15:02P *16:02P	−	−	*01:01P *01:01P	*01:02P *01:02P	*05:02P *05:02P	*02:02P *02:02P	*01:01P *05:01P
8	*02:05P *68:01P	*07:02P *52:03P	*03:03P *07:02P	*04:05P *14:06P	*01:01P	*01:01P	−	*03:01P *05:01P	*03:01P *03:02P	*01:03P *02:22	*03:01P *03:01P
9	*01:01P *02:01P	*08:01P *44:03P	*06:02P *16:01P	*03:01P *07:01P	*01:01P	*01:01P	−	*02:01P *05:01P	*02:01P *02:01P	*01:03P *02:01P	*02:01P *11:01P
10	*03:01P *31:01P	*08:01P *39:08	*07:01P *07:02P	*03:01P *04:07P	*01:01P	*01:01P	−	*03:01P *05:01P	*02:01P *03:02P	*01:03P *02:01P	*01:01P *04:02P
11	*02:01P *02:06P	*48:01P *51:01P	*08:01P *15:02P	*04:04P *04:11P	−	*01:01P *01:01P	−	*03:01P *03:01P	*03:02P *04:02P	*01:03P *01:03P	*04:02P *04:02P

Nine subjects responded to the SARS‐CoV‐2 peptide megapools with some differences (Fig. 1). Five subjects (#1, 2, 6, 8, 10) showed concurrent CD4+ T cell and CD8+ T‐cell responses to SARS‐CoV‐2 and CCC (Fig. 1). These patients differed in disease severity and there was no clear relationship between the magnitude or character of their T‐cell response and their clinical course. Subjects #1, 2, and 10 were treated on the ward and did not require inotropic support. In contrast, subjects #6 and 8 had a severe form of MIS‐C and required inotropic support in the intensive care unit.

Subjects #1, 2, and 8 responded to both spike and nonspike peptide epitopes of CCC and Subject #6 responded only to CCC spike peptide epitopes. These results could be reflective of previous exposure to CCC and/or cross‐recognition between SARS‐CoV‐2 and CCC epitopes. CD4+ T cells from Subject #10 did not recognize CCC peptide epitopes.

Three subjects had a CD4+ but no CD8+ T‐cell response. CD4+ T cells from Subjects #3 and 11 recognized SARS‐CoV‐2 spike and nonspike peptide epitopes and CD4+ T cells from Subject #4 recognized only SARS‐CoV‐2 spike peptide epitopes. With respect to the CCC responses, CD4+ T cells from Subjects #4 and 11 recognized spike and nonspike peptide epitopes and CD4+ T cells from Subject #3 only recognized spike peptide epitopes. Clinically, these three subjects had less severe MIS‐C with no requirement for inotropic support. Subject #3 (8 yo Caucasian female) and Subject #4 (7 yo Hispanic male) both had mild MIS‐C with normal echocardiograms, Subject #11 (16 yo Hispanic female) had more severe MIS‐C with a low normal left ventricular ejection fraction of 56% and a dilated left anterior descending coronary artery with a *Z* score of 3.5. All recovered with no sequelae.

The HLA class I and class II typing of these subjects revealed no specific pattern and did not differentiate subjects with coordinated CD4+ and CD8+ T‐cell responses. One of 11 subjects, #5, a 5 yo Latina female, showed only a CD8+ T‐cell response and had mild disease.

### Expression of memory markers and chemokine receptors in SARS‐CoV‐2‐specific T cells in MIS‐C

**Figure 2:**
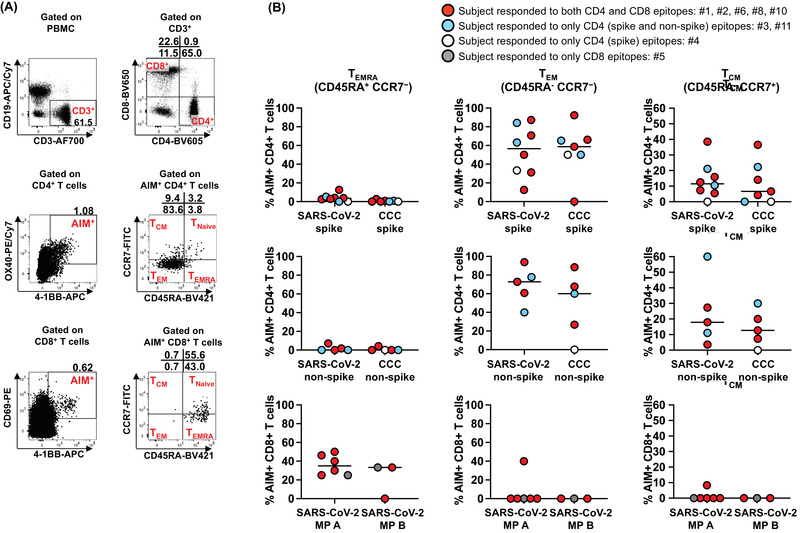
**Memory phenotypes of antigen‐specific, AIM+ CD4+ and CD8+ T cells in MIS‐C subjects**. Memory phenotype of the antigen‐specific AIM+ CD4+ and CD8+ T cells were studied 24 h after the stimulation. **(A)** Gating strategies to study CD4+ and CD8+ terminally differentiated effector T cells (T_EMRA_), effector (T_EM_) and central (T_CM_) memory T cells. T_EMRA_ was defined as CD45RA+ CCR7‐. Memory T cells, CD45RA‐ were further characterized by the expression of CCR7 as T_EM_ (CD45RA‐ CCR7‐) and T_CM_ (CD45RA‐ CCR7+). **(B)** Antigen‐specific CD4+ and CD8+ T_EMRA_, T_EM_, and T_CM_ in MIS‐C subjects. Each symbol shows the percentage of T_EMRA_ (left panels), T_EM_ (middle panels), and T_CM_ (right panels) in the AIM+ CD4+ or CD8+ T‐cell populations. Red circles: subjects responding to both CD4 and CD8 epitopes (#1, 2, 6, 8, and 10); blue circles: subjects responding to only CD4 (spike and nonspike) epitopes (#3 and 11); white circle: subjects responding to only CD4 (spike) epitopes (#4); gray circle: subjects responding to only CD8 epitopes (#5). Symbols represent the data derived from each individual subject. The study of 11 subjects was completed in seven independent experiments (one or two subjects/experiment depending upon enrollment). Medians were calculated and reported in the figure. Antigen‐specific CD8+ T cells showed a higher T_EMRA_ phenotype than CD4+ T cells. In contrast, T_EM_ and T_CM_ were more prevalent in antigen‐specific, AIM+ CD4+ T cells than CD8+ T cells.

Next, we characterized SARS‐CoV‐2‐specific terminally differentiated effector T cells (T_EMRA_), effector memory T cells (T_EM_), and central memory T cells (T_CM_) by measuring CD45RA and CCR7 expression on AIM+ T cells (Supporting information Fig. S1A). As expected, most responding SARS‐CoV‐2‐specific CD4+ T cells were of the T_EM_ and T_CM_ subsets, and low numbers of T_EMRA_ cells were detected (Fig. 2). By contrast, in the case of SARS‐CoV‐2‐specific CD8+ T cells, high numbers of T_EMRA_ cells were detected with low numbers of T_EM_ and T_CM_ (Fig. 2).

To further characterize the responding T‐cell populations, we also tested the expression of the chemokine receptor CCR6 on AIM+ T cells. CCR6 binds CXCL20 expressed on the endothelial side of the vessels, lungs, and the mucosal side of the gut and defines potential T‐cell homing to these compartments [11, 12, 13]. CCR6 was expressed on both spike‐specific and nonspike‐specific CD4+ T cells (31.7–76.3%, median 55.4% and 56.4–80%, median 64.7%, respectively) (Fig. 1A, right panels). The expression of CCR6 was significantly lower in CD8+ T cells (0–20%, median 0%) (Fig. 1B, right panels; *p* < 0.0001 as compared to CD4 T cells, Supporting information Fig. S3A). No statistical differences were found in terms of CCR6 expression on CCC‐specific T cells in the same subjects (*p* > 0.05). To better understand if the CCR6 expression on T cells was suggestive of homing to the gut and/or to the vessels and lungs, we studied the coexpression of the chemokine receptor CCR9 that determines homing to the gut. The results in Supporting information Fig. S3, panels B and C show that the majority of SARS‐CoV‐2‐specific T cells expressed CCR6 and did not express CCR9, with low percentages of double‐positive T cells and T cells expressing only CCR9.

### T‐cell response to SARS‐CoV‐2 and CCC in convalescent post‐COVID‐19 children and adults

**Figure 3:**
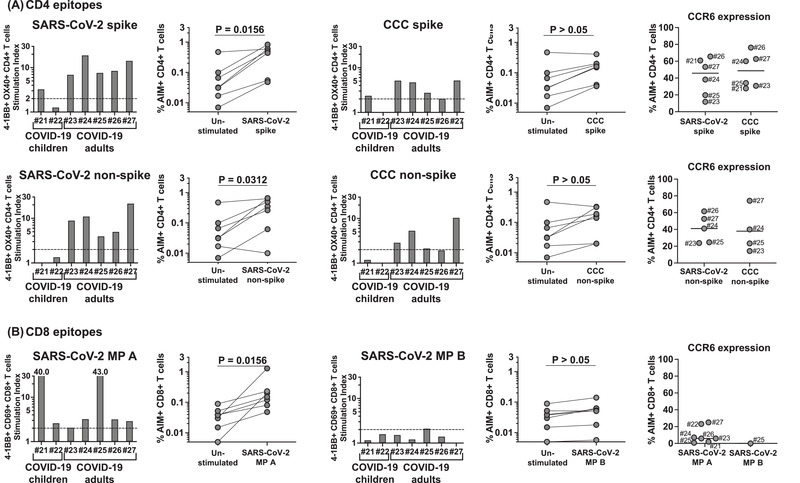
**CD4+ and CD8+ T‐cell responses to SARS‐CoV‐2 and CCC peptide megapools in convalescent SARS‐CoV‐2‐infected children and adults**. CD4+ and CD8+ T‐cell responses to SARS‐CoV‐2 and CCC were tested on two convalescent SARS‐CoV‐2‐infected children (#21‐22, 84 and 103 days after disease onset, respectively) and on five convalescent SARS‐CoV‐2‐infected adult subjects (#23–27, 100 to 146 days after disease onset). **(A)** CD4+ T‐cell responses and CCR6 expression to CD4 peptide megapools in each convalescent SARS‐CoV‐2‐infected subject. One convalescent SARS‐CoV‐2‐infected child (#21) and all five convalescent SARS‐CoV‐2‐infected adults (#23 ‐ 27) showed CD4+ T‐cells responses to the CD4 megapools derived from both SARS‐CoV‐2 and CCC. CCR6 expression on CD4+ T cells from convalescent SARS‐CoV‐2 infected subjects was similar to the MIS‐C subjects. **(B)** CD8+ T‐cell responses and CCR6 expression to CD8 peptide megapools in each convalescent SARS‐CoV‐2‐infected subject. Symbols represent the data derived from each individual subject. Seven subjects were studied in four independent experiments (one to three subjects/experiment depending upon enrollment). All the convalescent SARS‐CoV‐2‐infected subjects showed CD8+ T‐cell responses to at least one of the SARS‐CoV‐2 CD8 megapools. Low percentages of AIM+ CD8+ T cells expressed CCR6 as found in MIS‐C. Comparisons of the percentage of AIM+ T cells in the unstimulated control and peptide megapool‐stimulated cultures were tested by Wilcoxon signed rank test.

As a comparator, we enrolled seven subjects who had recovered from acute SARS‐CoV‐2 infection 3 to 5 months prior to the study (Table 3). The results shown in Fig. 3 suggested similar patterns of T‐cell recognition in these controls as compared to MIS‐C and the magnitude of the response, expressed as a stimulation index (SI) was not significantly different (*p* > 0.05). One of the convalescent children, #21, showed a CD4+ T‐cell response to spike SARS‐CoV‐2 and CCC and a CD8+ T‐cell response (Fig. 3). The second child, #22, had only a CD8+ T‐cell response. In all five adults, we detected both CD4+ and CD8+ T‐cell responses. Only in one subject, #26, CD4+ T cells did not recognize nonspike peptides. The response to CCC was also appreciable.

### Enumeration of memory SARS‐CoV‐2‐specific T cells in convalescent children and adults

**Figure 4:**
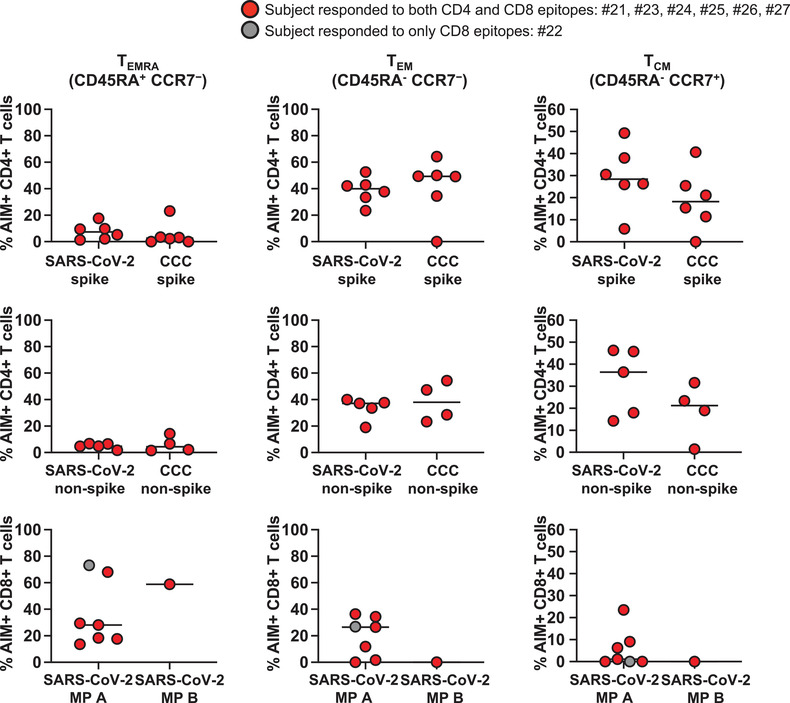
**Memory phenotypes of antigen‐specific AIM+ CD4+ and CD8+ T cells in convalescent SARS‐CoV‐2‐infected children and adults**. T_EMRA_, T_EM_, and T_CM_ of the antigen‐specific AIM+ CD4+ and CD8+ T cells from the convalescent SARS‐CoV‐2‐infected subjects were enumerated 24 h after the stimulation. Each symbol shows the percentage of T_EMRA_ (left panels), T_EM_ (middle panels), and T_CM_ (right panels) in the AIM+ CD4+ or CD8+ T‐cell populations. Red circles: subjects responding to both CD4 and CD8 epitopes (#21, 23–27); gray circle: subjects responding to only CD8 epitopes (#22). Symbol represents the data derived from each individual subject. Seven subjects were studied in four independent experiments (one to three subjects/experiment depending upon enrollment). Medians were calculated and reported in the figure. T_EMRA_ were more numerous in antigen‐specific, AIM+ CD8+ T cells than in CD4+ T cells. T_EM_ and T_CM_ were more numerous in antigen‐specific, AIM+ CD4+ T cells than CD8+ T cells.

We then characterized SARS‐CoV‐2‐specific T_EMRA_, T_EM_, and T_CM_ on AIM+ T cells in the convalescent subjects (Fig. 4). Similar to MIS‐C, T_EMRA_ were low in AIM+ CD4+ T cells but high in AIM+ CD8+ T cells. In contrast, as previously reported in MIS‐C, AIM+ CD4+ T_EM_ and T_CM_ were significantly more numerous than CD8+ T_EM_ and T_CM_. There were no significant differences in the T‐cell memory phenotypes between MIS‐C and convalescent SARS‐CoV‐2‐infected subjects (*p* > 0.05).

Of interest, the expression of CCR6 on AIM+ T cells was similar to the MIS‐C patients (*p* > 0.05). On CD4+ spike‐specific T cells, the percentage of CCR6 expression ranged from 11.8 to 65.6% (median 45.8%), on nonspike‐specific CD4+ T cells CCR6 expression ranged between 23.8 and 61.6% (median 41.1%) and on CD8+ T cells CCR6 expression ranged between 0 and 25% (median 5.9%) (Fig. 3, right panels). The CCR6 expression on AIM+ CD4+ T cells was higher than the CCR6 expression on AIM+ CD8+ T cells in convalescent SARS‐CoV‐2‐infected subjects as previously observed in MIS‐C (*p* < 0.0001) (Supporting information Fig. S3A). The results in Supporting information Fig. S3, panels B and C show that, as detected in the case of MIS‐C subjects, the majority of SARS‐CoV‐2‐specific T cells in convalescent SARS‐CoV‐2‐infected subjects expressed CCR6 and did not express CCR9, with low percentages of double‐positive T cells and some expressing only CCR9.

### Reactivity to SARS‐CoV‐2 and CCC in KD subjects enrolled before and during the pandemic

MIS‐C and KD share some clinical similarities but are two distinct syndromes defined by differences in inflammatory parameters, nature of the cardiac manifestations, and exposure to SARS‐CoV‐2. As a control to address the role of the CCC T‐cell response in the MISC cohort in shaping SARS‐CoV‐2‐specific T‐cell responses, we studied SARS‐CoV‐2 and CCC‐specific T cells in 10 KD subjects, five enrolled before the SARS‐CoV‐2 pandemic and five enrolled during the pandemic (Table 4).

**Table 3:** Convalescent SARS‐CoV‐2‐infected pediatric and adult subjects enrolled in this study

Subjects	Age (yrs)	Sex	Illness day at symptom onset
21	11	M	103
22	14	M	89
23	29	F	119
24	63	M	146
25	39	M	100
26	67	F	132
27	52	M	119

**Table 4:** KD subjects enrolled in this study

Subjects	Age (yrs)	Sex	Illness day at diagnosis	Illnessday at phlebotomy	Zmax^a^	WBC (103/μL)	Hgb (g/dL)	Neutrophils (%)	Bands (%)	ESR (mm/h)	CRP (mg/dL)	ALT (IU/L)	GGT (IU/L)	Aboslute lymphocyte count
28	1.4	F	4	7	2.49	12.5	11	43	33	65	19.4	87	53	1750
29	5.8	F	5	6	0.6	9.6	9.9	58	7	8	8.3	23	55	2400
30	1.9	F	7	7	2.97	19.3	9.7	52	21	77	33.4	27	108	3281
31	1.9	F	7	7	2.69	16.7	10	65	14	127	14.4	48	29	2171
32	8.8	M	4	36	1.78	7.6	11.5	33	63	32	4.4	139	121	152
33	3.0	F	7	26	1.7	19.2	10.1	65	3	61	4.5	67	28	4608
34	7.1	M	3	27	0.1	15.4	13.3	51	19	34	6.1	104	98	1078
35	8.9	M	10	29	1.46	9.9	12	58	0	38	3.1	13	18	3564
36	6.6	F	4	46	1.07	18.3	12.4	74	3	30	2.5	113	170	1830
37	5.6	F	6	43	1.5	17.5	12.5	73	6	75	3.4	204	161	1750
38	7.0	M	16	38	2.02	15.6	10.5	69	3	58	4.4	93	656	1248
39	9.1	M	4	18	0.15	18	12.1	78	1	66	6.3	333	365	1800
40	9.1	M	5	27	1.33	7.8	12.4	53	2	47	0.6	80	145	3042
41	4.2	F	7	22	0.57	14.2	11.3	79.1	NA	53	22	197	235	1349
42	6.1	F	6	22	2.91	17.7	11.6	17	78	140	19.4	140	148	885
43	8.1	M	7	23	2.03	11.8	12.5	68	15	32	2.9	226	362	354
44	3.5	M	6	36	0.81	15.5	10.5	71	9	56	19.2	157	72	1705
45	2.3	M	11	15	3.12	11	10.8	63.3	NA	76	7.6	15	11	2453

^a^Zmax: maximum Z score (internal diameter normalized for body surface area) for the right and left anterior descending coronary arteries.

^b^Laboratory data are pretreatment.

*Abbreviations*: ALC, absolute lymphocyte count; ALT, alanine aminotransferase; CRP, C‐reactive protein; ESR, erythrocyte sedimentation rate; GGT, gamma‐glutamyl transferase; Hgb, hemoglobin; WBC, white blood cell count; NA, not available.

Subjects 28–32 and 38–45 were sampled before the pandemic. Subjects 33–37 were sampled during the pandemic but had negative antibody and nucleic acid testing for SARS‐CoV‐2 at the time of phlebotomy.

**Figure 5:**
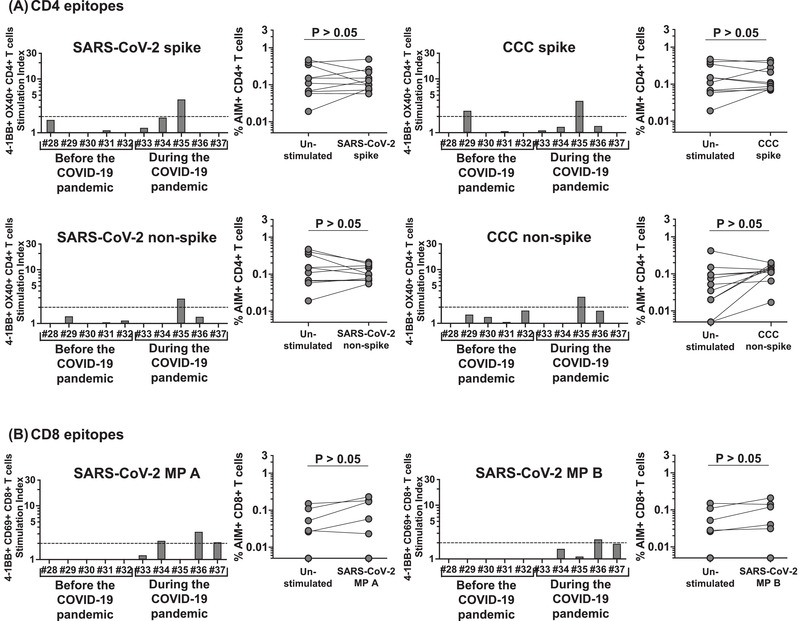
**CD4+ and CD8+ T‐cell responses to SARS‐CoV‐2 and CCC peptide megapools in KD children sampled before and during the COVID‐19 pandemic**. CD4+ and CD8+ T‐cell responses to SARS‐CoV‐2 and CCC were tested in five KD subjects (#28–32) sampled before the COVID‐19 pandemic (2011–2016) and in five KD subjects (#33–37) sampled during the COVID‐19 pandemic (May–July, 2020). **(A)** CD4+ T‐cell responses to CD4 peptide megapools in each individual KD subject. Four (#28, 30–32) of the five KD subjects sampled before the pandemic showed no responses to any of the CD4 megapools derived from SARS‐CoV‐2 or CCC, and one (#29) responded only to CCC CD4 spike. Similarly, four (#33, 34, 36, 37) of the five KD subjects enrolled during the pandemic showed no responses to any of the CD4 megapools derived from SARS‐CoV‐2 or CCC, and one (#35) showed concurrent responses to CD4 spike and nonspike from both SRAS‐CoV‐2 and CCC. **(B)** CD8+ T‐cell responses to CD8 peptide megapools in each individual KD subject. None of the KD subjects enrolled before the pandemic showed detectable response to SARS‐CoV‐2 CD8 megapools. Symbols represent the data derived from each individual subject. Ten subjects were studied in three independent experiments (two to five subjects/experiment depending upon enrollment). Three (#35–37) of the KD subjects enrolled during the pandemic showed a low response to SARS‐CoV‐2 CD8 megpaools. Comparisons of the percentage of AIM+ T cells in the unstimulated control and peptide megapool‐stimulated cultures were tested by Wilcoxon rank sign test.

None of the five KD subjects enrolled before the pandemic responded to SARS‐CoV‐2 peptide epitopes (Fig. 5). One of five subjects (Subject #29, Table 4), showed a mild response to the CCC spike megapool, likely due to CCC exposure. This patient was a 5.8‐year‐old female with a normal echocardiogram. Of the five KD subjects enrolled during the pandemic, one (#35), showed a CD4+ and CD8+ T‐cell response to SARS‐CoV‐2 and a CD4+ T‐cell response to CCC spike and nonspike megapools (Fig. 5). This was an 8.9‐year‐old boy who was the oldest in the cohort. The SARS‐CoV‐2‐specific T‐cell response was possibly due to cross‐reactive CCC‐specific T cells, but an asymptomatic antecedent exposure to SARS‐CoV‐2 cannot be ruled out.

**Figure 6:**
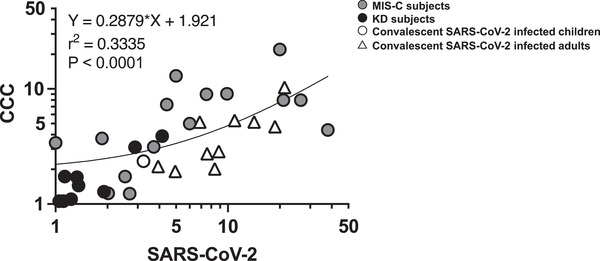
**Correlation of CD4+ T‐cell responses to SARS‐CoV‐2 and CCC**. The correlation of the SI from the individual subject in response to CD4 spike megapools derived from SARS‐CoV‐2 versus CCC and to CD4 nonspike megapools derived from SARS‐CoC‐2 versus CCC from all the cohorts were studied. Symbols represent the data derived from individual subjects including MIS‐C, convalescent SARS‐CoV‐2‐infected, and KD subjects with a total of 28 subjects studied in 14 independent experiments. A strong correlation of the CD4+ T‐cell responses was found between SARS‐CoV‐2 and CCC (*p* < 0.0001) suggesting a cross‐reactivity of the T‐cell response between SARS‐CoV‐2 and CCC and/or a previous exposure of CCC of the subjects. Pearson's correlation test was used. Circles: pediatric subjects; triangles: adult subjects; gray symbols: MIS‐C; black symbols: KD; white symbols: convalescent SARS‐CoV‐2‐infected subjects.

A significant correlation was detected in terms of the SI in response to CD4 spike megapools derived from SARS‐CoV‐2 versus CCC (Fig. 6) and to CD4 nonspike megapools derived from SARS‐CoV‐2 versus CCC from individual subjects in the MIS‐C and SARS‐CoV‐2 convalescent and KD cohorts.

### Immune phenotype of the APCs in MIS‐C and KD

Next, we enumerated monocytes, macrophages, and myeloid DCs that include cDC1, CD14+ cDC2, CD14‐ cDC2, pediatric CD4+ ILT‐4+ tolerogenic DC (tmDC) (Supporting information Fig. S1B) in eight additional MIS‐C and eight KD subjects enrolled before the SARS‐CoV‐2 pandemic to investigate the status of the APC and the innate immune tolerance in the subacute phase of MIS‐C and KD.

**Figure 7:**
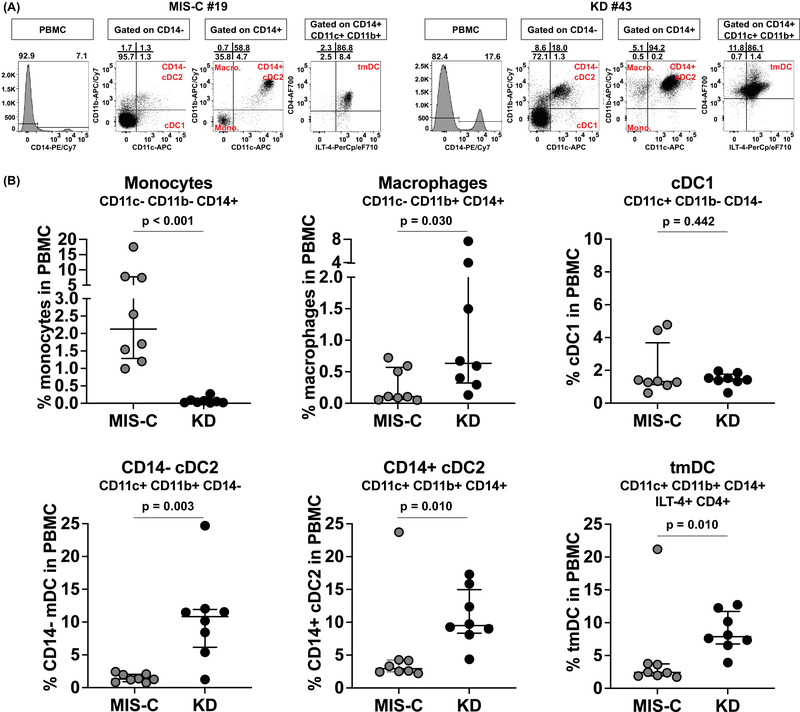
**Enumeration of innate APC in the PBMC from MIS‐C and KD subjects**. Eight MIS‐C subjects (#13–20) and eight subacute KD subjects (#38–45) were enrolled to study their innate APC populations by staining the PBMC with a combination of monoclonal antibodies. Monocytes (CD11c‐ CD11b‐ CD14+), macrophages (CD11c‐ CD11b+ CD14+), cDC1 (CD11c+ CD11b‐ CD14‐), CD14‐ cDC2 (CD11c+ CD11b+ CD14‐), CD14+ cDC2 (CD11c+ CD11b+ CD14+), and tmDC (ILT‐4+ CD4+ CD14+ cDC2) were enumerated from each subject. **(A)** Representative FACS plots showing the gating of different innate APC populations from one MIS‐C subject (#19) and one subacute KD subject (#43). **(B)** Percentage of each innate APC population in the PBMC from the eight MIS‐C (grey) and eight subacute KD subjects (black). Symbols represent the data derived from each individual subject. A total of 16 subjects were studied in ten independent experiments. Median ± interquartile ranges are indicated in the figure. Comparisons of the percentage of each innate APC population between subacute MIS‐C and KD subjects were tested by Mann–Whitney U tests. MIS‐C subjects showed a higher percentage of monocytes (*p* < 0.001) but lower percentages of macrophages (*p* = 0.03), CD14‐ cDC2 (*p* = 0.003), CD14+ cDC2 (*p* = 0.01), and tmDC (*p* = 0.01) than KD subjects.

In KD, tmDC are of the most important in controlling the immune homeostasis [14], they contribute to the successful response to intravenous immunoglobulin (IVIG) therapy and play a role in the clinical presentation [15]. In MIS‐C, the status of the innate compartment and the extent of activation of tolerogenic CD14+ cDC2 and tmDC could have played a significant role in the disease pathogenesis. The results, shown in Fig. 7, revealed sharp differences between MIS‐C and KD. The most important observation was a significantly low cell number in MIS‐C compared to KD for the following populations: CD14‐ cDC2 (canonical APC for T‐cell presentation), (*p* = 0.003), CD14+ cDC2 with tolerogenic phenotype (*p* = 0.010), and tmDC (*p* = 0.010). Macrophages were also lower in MIS‐C compared to KD (*p* = 0.030). Conversely, monocyte precursors were higher in MIS‐C (*p* < 0.001) suggesting active myelopoiesis in the BM (Fig. 7).

## Discussion

MIS‐C develops in a small percentage of children who have been exposed to SARS‐CoV‐2. This study addresses the T‐cell recognition of viral epitopes in MIS‐C, the development of SARS‐CoV‐2‐specific T‐cell memory, and the contribution of cross‐reactivity to CCC to SARS‐CoV‐2‐specific T‐cell responses. Our results suggest that the magnitude of the T‐cell response in MIS‐C is similar to the convalescent response following SARS‐CoV‐2 infection in children and adults. The differences in the CD4+ Th response and the CD8+ CTL response were also comparable in the two cohorts.

Nine of 11 of the children with MIS‐C responded to SARS‐CoV‐2 peptide epitopes, but the patterns did not correlate with either disease severity or HLA type. The HLA class I and class II characterization of the five MIS‐C subjects who had both CD4+ and CD8+ T‐cell responses did not explain the differences with other subjects in the cohort who mounted only a CD4+ T‐cell response. A recent hypothesis that MIS‐C disease severity is linked to HLA A2, HLA B35, and HLA C4 that are capable of presenting superantigens derived from the SARS‐CoV‐2 spike glycoproteins to specific oligoclonal TCRs carrying the V beta 11.2 chain [16] was not supported by our data. There was also no association of HLA type with disease severity. In our cohort, of the four subjects carrying the HLA A2 allele, only Subject 8 required intensive care. Of the two subjects carrying HLA B35 and three subjects carrying HLA C4, all had mild disease and none required intensive care.

It has been suggested that in MIS‐C patients, the T‐cell response is compromised and that a specific oligoclonal expansion of V beta 21.3 T cells defines the T‐cell repertoire [17]. Our data on the SARS‐CoV‐2‐specific T‐cell response neither suggest a defect in antiviral‐specific T cells nor were there differences in the virus‐specific T‐cell responses in the children with MIS‐C compared to convalescent controls following SARS‐CoV‐2 infection. In our MIS‐C cohort, the T‐cell response to the SARS‐CoV‐2 and CCC megapools was similar in magnitude, which could be consistent with either previous exposure to CCC or cross‐reactivity between coronaviruses as defined in adults [9]. The two youngest subjects showed no detectable SARS‐CoV‐2‐specific T‐cell response. The HLA typing of these subjects showed shared alleles with others in the cohort who made an appropriate T‐cell response to SARS‐CoV‐2 peptides.

Chemokine receptors are important for T‐cell homing [11, 12, 13]. In our study, a high percentage of T cells, especially CD4+ T cells, expressed CCR6, which suggests trafficking to the endothelium, lungs, and gut that express CXCL20, the ligand for CCR6. Only few AIM+ T cells coexpressed CCR9, expressed by tissue‐resident T cells in the ileum and colon, suggesting that proinflammatory SARS‐CoV‐2‐specific T cells.

Specific markers define antigen‐specific memory T cells in humans and the role of CD4+ T cells within the development of memory CD8+ T cells [18, 19, 20, 21, 22]. In the present study, differences in the development of SARS‐CoV‐2‐specific T‐cell memory were observed with abundant effector and central memory T cells within only the CD4+ but not in CD8+ T‐cell populations. Terminally differentiated effector T cells were abundant within the SARS‐CoV‐2‐specific CD8+ but not in CD4+ T cells that suggests either differences in the timing of memory development or the possibility of viral persistence [23]. The possibility of viral persistence, although not proven, is an appealing hypothesis in MIS‐C, where we found a low number of innate APC precursors suggesting exhaustion caused by the persistence of the antigen [26]. We also found very low tolerogenic cDC2 and tmDC. The low number of cells responsible for the innate immune regulation in MIS‐C and the sharp differences in the enumeration and activation stage of these cells between MIS‐C and KD suggest a possible role for the lack of innate immune regulation in the pathogenesis of MIS‐C.

The KD cohort was included in the analysis as an ideal control cohort of similarly aged children with acute and subacute inflammation. We also took this opportunity to explore the hypothesis that prior exposure to CCC could result in delayed immune activation analogous to what is seen in MIS‐C. However, only two of the 10 KD subjects had T cells that responded to CCC. The T cells of one KD subject enrolled during the pandemic responded to both CCC and SARS‐CoV‐2 peptide epitopes suggesting either cross‐reactivity or subclinical exposure to SARS‐CoV‐2.

The immune phenotype of the innate APC compartment in MIS‐C suggested very low numbers of myeloid cells in sharp contrast with KD that include classical APC for T‐cell presentation CD14‐ cDC2, tolerogenic CD14+ cDC2 and tmDC. Reversely, monocytes were found much higher in MIS‐C than suggesting active myelopoiesis concomitant to low mature DC in circulation that is usually associated with viral persistence. Moreover, our previous work pointed to an important role for pediatric tmDC in reducing the inflammation in KD [14]: lack of tmDC in MIS‐C could also contribute to the inflammatory process in these patients.

We recognize both strengths and limitations of our study. We present a comprehensive characterization of the T‐cell response to SARS‐CoV‐2 that includes a detailed clinical description of disease severity in the MIS‐C patients, coupled with HLA typing. We also explored the expression of chemokine receptors on SARS‐CoV‐2‐specific T cells that define homing to the endothelial compartment and to the gut. A limitation is the lack of access to tissues to better define T‐cell trafficking. Limitations in cell numbers did not allow for a comprehensive characterization of the T‐cell response to SARS‐CoV‐2, including potential alteration in the cytokine pattern and functionality of the responding T cells, and the definition of the specific epitopes recognized (as opposed to recognition of the peptide pools utilized herein). The number of subjects analyzed was limited, and as such, the conclusion that no differences were detected should be considered as a preliminary observation. The CCC data are consistent with but does not establish cross‐reactivity. The MIS‐C children have been presumably infected, independently, with CCC and SARS‐CoV‐2, and therefore, detection of both CCC and SARS‐CoV‐2 reactivity is consistent but do not prove that the reactivity is mediated by the same cross‐reactive cells. The observation that certain HLA alleles in MIS‐C children were shared by other individuals in the cohort who did respond to SARS‐CoV‐2 peptides, thus, suggesting that their lack of response was not due to HLA restriction could also reflect that the subjects responding to SARS‐CoV‐2 may be using the alleles that are not shared with the MIS‐C.

In summary, SARS‐CoV‐2‐infected children who subsequently develop MIS‐C showed different patterns of T‐cell responses to SARS‐CoV‐2 peptide epitopes and cross‐reactivity to CCC that did not correlate with age, clinical severity, or HLA type. Reduced numbers of CD14+ cDC2 and tmDC may contribute to the hyperinflammatory state in these patients.

## Materials and methods

### Study populations

The study protocol for MIS‐C and KD subjects was approved by the Institutional Review Board at the University of California San Diego (IRB #140220). Subjects were enrolled at Rady Children's Hospital, San Diego, following written parental informed consent and patient assent as appropriate. Eleven MIS‐C subjects, seven males and four females aged 8 months to 16 years were enrolled in the study from May to September 2020, 15–52 days after MIS‐C onset to study SARS‐CoV‐2 and CCC T‐cell responses. One additional MIS‐C subject (male, 10.4 years old), was enrolled to determine the expression of chemokine receptors on SARS‐CoV‐2‐specific T cells. Eight additional MIS‐C subjects, three males and three females aged 3.7 to 12.9 years were enrolled in the study of the immune phenotype of the innate cells. Clinical and laboratory data from MIS‐C patients at the time of hospital admission are described in Table 1. SARS‐CoV‐2 exposure was determined by PCR and antibody measurement. Only one subject, #3, was clinically a MIS‐C (confirmed by our results that indicated a SARS‐CoV‐2‐specific T‐cell response) but PCR and antibody negative. Blood samples were collected from MIS‐C subjects following IVIG and other anti‐inflammatory treatments 15–52 days after fever onset. Blood samples were also collected from seven SARS‐CoV‐2‐infected convalescent controls: 2 male children aged 11 and 14 years, the study protocol was approved by the Institutional Review Board at the University of California San Diego (IRB #200493) and five adults (three males and two females) aged 29 to 67 years, the study protocol was approved by the Institutional Review Board at La Jolla Institute for Immunology (#VD‐214) through the CRO‐BioIVT, studied 3 to 5 months after symptom onset (Table 3). For the 18 KD subjects, coronary artery status was defined as *Z*max: maximum *Z* score (internal diameter normalized for body surface area) for the right and left anterior descending coronary arteries (Table 4). SARS‐CoV‐2 and CCC T‐cell responses were tested in subjects #28‐37. Of these KD subjects, five (#28–32) were enrolled before the COVID‐19 pandemic (2011 to 2016; two males, three females aged 2.9–8.9 years), and five (#33–37) were enrolled during the COVID‐19 pandemic (May to July 2020, four females, one male aged 1.4 to 7.8 years). Eight KD subjects (#38–45), six males and two females, aged 4 to 16 years were enrolled to study the innate cell compartment and its activation. Blood samples from KD subjects were collected 6–46 days after fever onset.

### Peptide megapools

Two SARS‐CoV‐2 CD4 megapools, two SARS‐CoV‐2 CD8 megapools, and two CCC CD4 megapools were used to study the CD4+ and CD8+ T‐cell responses to SARS‐CoV‐2 and CCC in MIS‐C, KD, and SARS‐CoV‐2‐infected control subjects. The megapools were designed based on the reference genomic sequence of Wuhan‐Hu‐1 SARS‐CoV‐2 isolate (GenBank ID:MN908947), as described and validated in acute and convalescent SARS‐COV‐2‐infected patients, as well as unexposed healthy subjects [5, 6, 24]. The SARS‐CoV‐2 CD4 spike megapool contains 253 15‐amino acid‐long peptides overlapping 10 amino acids and spanning the entire spike protein. The SARS‐CoV‐2 CD4 nonspike megapool contains 221 15‐mers predicted HLA class II epitopes derived from the remainder (nonspike) of the SARS‐CoV‐2 proteome. The two SARS‐CoV‐2 CD8 megapools contain a total of 628 peptides (314 in each megapool), predicted to bind 12 HLA A & B most frequent alleles in the general human population (A*01:01, A*02:01, A*03:01, A*11:01, A*23:01, A*24:02, B*07:02, B*08:01, B*35:01, B*40:01, B*44:02, B*44:03). The CCC spike and nonspike megapools contain 124 and 129 peptide epitopes‐derived from the four CCC (229E, NL63, OC43, and HKU1), which are homologs of immunodominant SARS‐CoV‐2 epitopes identified in unexposed healthy donors as previously described [6]. Peptides were synthesized as crude material (T.C. Laboratories, San Diego, CA), resuspended in DMSO, pooled according to megapool design and allowed by sequential relyophilization [25].

### Activation‐induced markers (AIM) assay

PBMC were separated from heparinized whole blood from MIS‐C, convalescent SARS‐CoV‐2‐ infected and KD subjects by Ficoll–Hypaque density centrifugation and frozen in liquid nitrogen. After thawing, 1 × 10^6^ cells were stimulated in 96 wells U bottom plates with 1 microgram per milliliter of different peptide megapools. PBMC cultured with 0.1% DMSO, the same concentration of DMSO (solvent) in the megapool‐stimulated cultures, served as unstimulated controls. Twenty four hours later, cell cultures were harvested and stained with monoclonal antibodies to be analyzed by flow cytometry [26] to study T‐cell activation, CCR6 and CCR9 expression, effector and memory phenotypes: anti‐CD3‐AF700 (clone OKT3, mouse IgG2aκ, BioLegend), anti‐CD4‐BV605 (clone RPA‐T4, mouse IgG1κ, BD Bioscience), anti‐CD8‐BV650 (RPA‐T8, mouse IgG1κ, BioLegend), anti‐4‐1BB‐APC (clone 4B4‐1, mouse IgG1κ, BioLegend), anti‐OX40‐PE/Cy7 (clone Ber‐ACT35, mouse IgG1κ, Biolegened), anti‐CD69‐PE (clone FN50, mouse IgG1κ, BD Bioscience), anti‐CCR6‐PerCp/Cy5.5 (clone 11A9, mouse IgG1κ, BD Bioscience), anti‐CD45RA‐BV421 (clone HI100, mouse IgG1κ, BioLegend), and anti‐CCR7‐FITC (clone G043H7, mouse IgG2aκ, BioLegend). Data were recorded on LSRFortessa (BD Bioscience) and analyzed with FlowJo software version 10 (Tree Star). Isotype controls for each antibody were tested and showed no staining.

Antigen‐specific responses were determined by the expression of T‐cell AIM assay [24] by measuring the coexpression of 4‐1BB and OX‐40, two TNF family member costimulatory molecules upregulated following TCR signaling on CD4+ T cells, and by measuring the coexpression of 4‐1BB and CD69 (adhesion molecule involved in lymphocyte homing and trafficking) on CD8+ T cells. The expression of the chemokine receptor CCR6 on AIM+ CD4+ and CD8+ T cells was also analyzed. Terminally differentiated effector T cells (T_EMRA_, CD45RA+ CCR7‐), effector memory T cells (T_EM_, CD45RA− CCR7−), and central memory T cells (T_CM_, CD45RA− CCR7+) were enumerated on AIM+ CD4+ and CD8+ T cells. The gating strategy of AIM+ CD4+ and CD8+ T cells is shown in Supporting information Fig. S1A.

### Immune phenotyping of myeloid APC

Innate myeloid cells were defined by surface markers by staining with monoclonal antibodies and analyzed by flow cytometry gating on specific populations: anti‐human CD11c‐allophycocyanin, clone B‐ly6, mouse IgG1κ; anti‐human CD11b‐allophycocyanin/Cy7, clone ICRF44, mouse IgG1κ; anti‐human CD14‐PE/Cy7, clone M5E2, mouse IgG2aκ (BD Biosciences); anti‐human BDCA‐1‐PE/Dazzle594, clone L161, mouse IgG1κ (BioLegend); anti‐human ILT‐4‐PerCp/eF710, clone 42D1, rat IgG2aκ (eBioscience); anti‐human CD4‐AF700, clone RPA‐T4, mouse IgG1κ (BD Biosciences); anti‐human CD16‐BV605, clone B73.1, mouse IgG1κ. The activation/maturation of the innate immune cells that present antigen to T cells was defined by the expression of CD86 by using anti‐human‐CD86 FITC, clone FUN‐1, mouse IgG1κ (BD Biosciences). Data were acquired on BD CANTO II and analyzed with FlowJo software version 10 (Tree Star). The gating strategy for the immune phenotyping of myeloid APC is shown in Supporting information Fig. S1B.

### HLA typing

High‐resolution typing of *HLA, A, B, C, DRB1, DRB3/4/5, DQB1, DQA1, DPA1, and DPB1 *was determined by sequence‐specific oligonucleotide probe method (LabType kit, One Lambda, West Hills, CA) and/or by next generation sequencing (AllType NGS kit, One Lambda).

### Statistical analysis

Data were analyzed using Prism software version 9.0 (GraphPad Software). To compare the percentage of AIM+ T cells in the unstimulated control and peptide stimulation, data obtained from each peptide megapool‐stimulated culture and unstimulated controls in the individual cohort were tested using nonparametric paired tests. A *p* value of less than 0.05 was considered significant. Mann–Whitney U tests were used to compare the differences of CCR6 expression on AIM+ CD4+ and CD8+ T cells, the differences of CCR6 expressions and memory phenotypes on AIM+ CD4+ and CD8+ T cells from different cohorts, and the differences of different APC types between MIS‐C and KD subjects, a linear regression analysis was used to test the correlation of the CD4+ T‐cell responses to SARS‐CoV‐2 and CCC. A *p* value of less than 0.05 was considered significant.

## Conflict of interest

The authors declare no conflict of interest. LJI has filed for patent protection for various aspects of vaccine design and identification of specific epitopes.

## Author Contributions

Li‐En Hsieh, performed the experiments under the supervision of Alessandra Franco and contributed to the manuscript preparation. Alba Grifoni and John Sidney provided the SARS‐CoV‐2 peptide megapools and adult convalescent controls.

Chisato Shimizu and Hiroko Shike generated the HLA typing data; Nanda Ramchandar provided pediatric convalescent controls; Elizabeth Moreno bridged the Clinic to the lab by collecting samples and patient information; Adriana H. Tremoulet, and Jane C. Burns diagnosed and treated MIS‐C and KD children, Jane C. Burns also participated to the editing of the manuscript, Alessandra Franco designed, directed, supported the study and wrote the manuscript.

## Ethics approval statement for human studies

The study protocol for MIS‐C and KD subjects was approved by the Institutional Review Board at the University of California San Diego (IRB #140220). The study protocol for SARS‐CoV‐2 infected convalescent children was approved by the Institutional Review Board at the University of California San Diego (#IRB 200493) and for SARS‐CoV‐2 infected convalescent adults was approved by the Institutional Review Board at La Jolla Institute for Allergy and Immunology (#VD‐214) through the CRO‐BioIVT. The pediatric subjects were enrolled following written parental informed consent and patient assent as appropriate. The adult subjects were enrolled following written consent.

### Peer review

The peer review history for this article is available at https://publons.com/publon/10.1002/eji.202149556

**Abbreviations**
AIM: activation‐induced cell markers
CCC: common cold coronaviruses
CTL: cytotoxic T cell
KD: Kawasaki disease
IVIG: intravenous immunoglobulin
*MIS‐C*
: *Multisystem inflammatory syndrome‐in children*
SI: stimulation index
T_CM_
: central memory T cells
T_EM_
: effector memory T cells
T_EMRA_
: terminally differentiated effector T cells
tmDC: tolerogenic DC

## Supporting information

Supporting information

## Data Availability

The data that support the findings of this study are available from the corresponding author upon reasonable request.
