# Supplementary material for: Characterization of SARS‐CoV‐2 and common cold coronavirus‐specific T‐cell responses in MIS‐C and Kawasaki disease children
Source: Eur J Immunol. 2021 Oct 27;52(1):123–37. doi: 10.1002/eji.202149556 (PMC8646471; doi:10.1002/eji.202149556)
Supplement: Supplementary file 1 — Supporting information [file EJI-52-123-s001.pdf]

Supplementary Figure 1

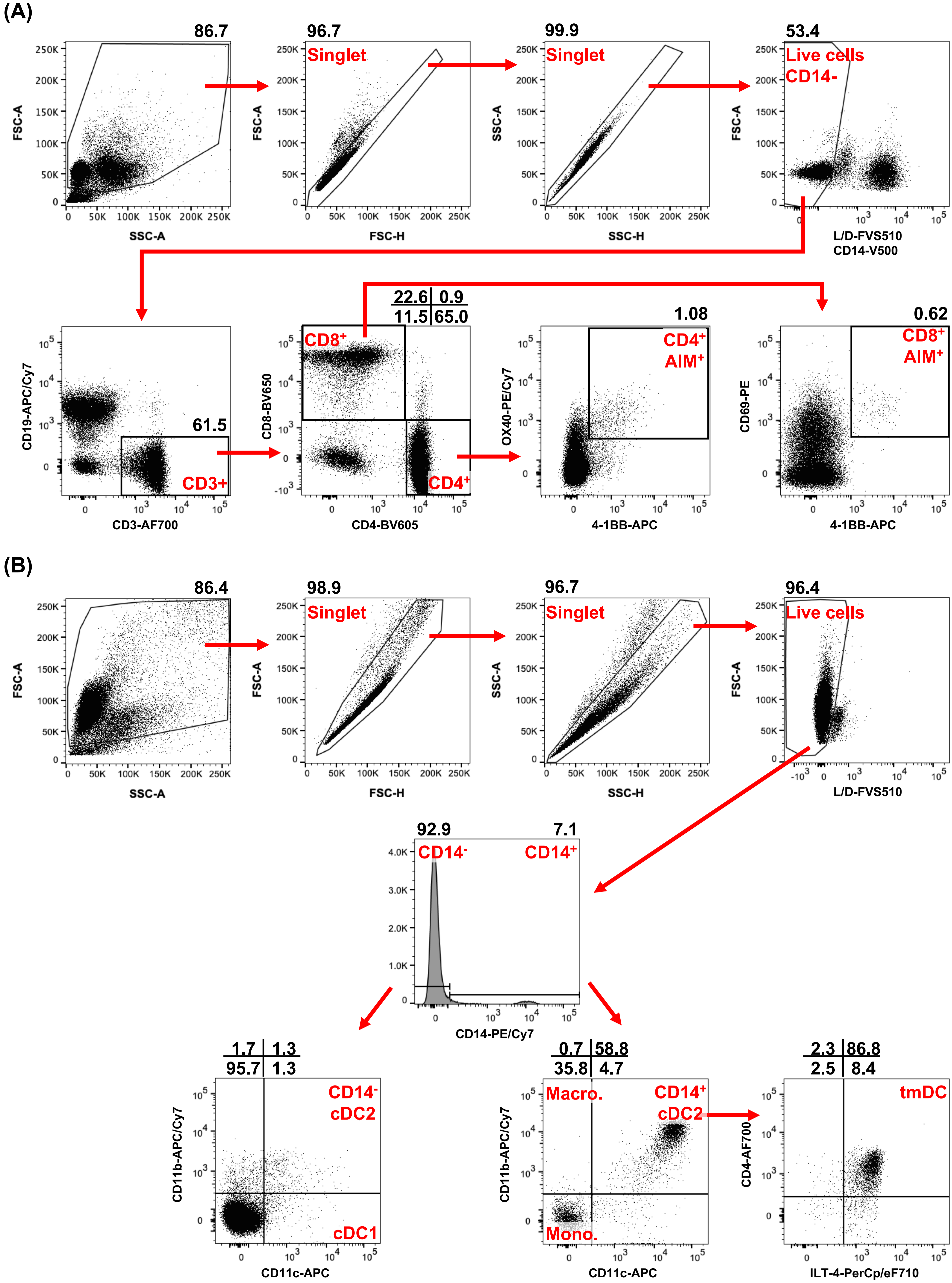

Supplementary Figure 1. Gating strategies to study SATS-CoV-2-specific CD4+ and CD8+ T cell (A) and myeloid cells in circulation (B) by flow cytometry.

Supplementary Figure 2

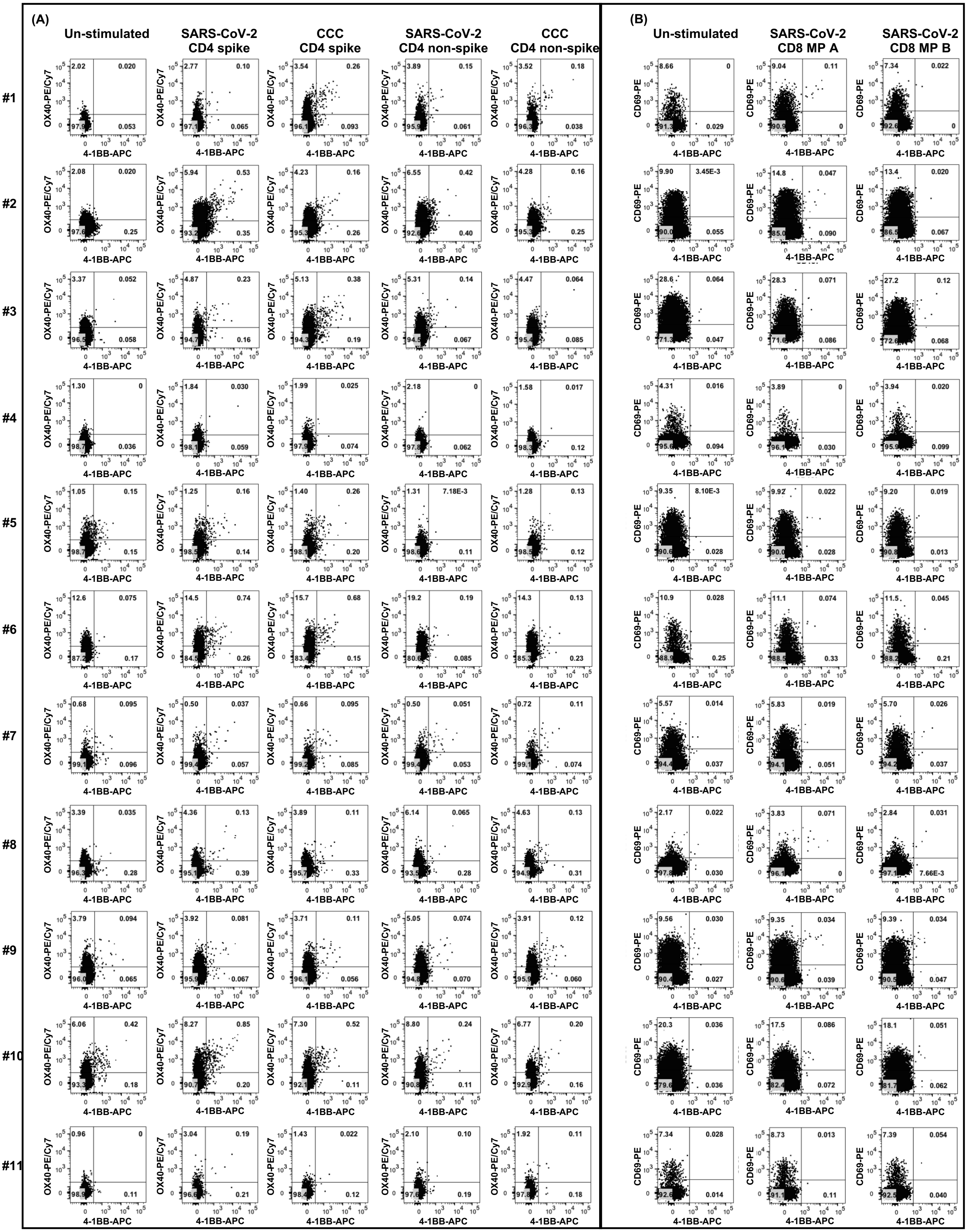

Supplementary Figure 2. AIM assay in MIS-C. Flow cytometry results in 11 MIS-C subjects.

Supplementary Figure 3

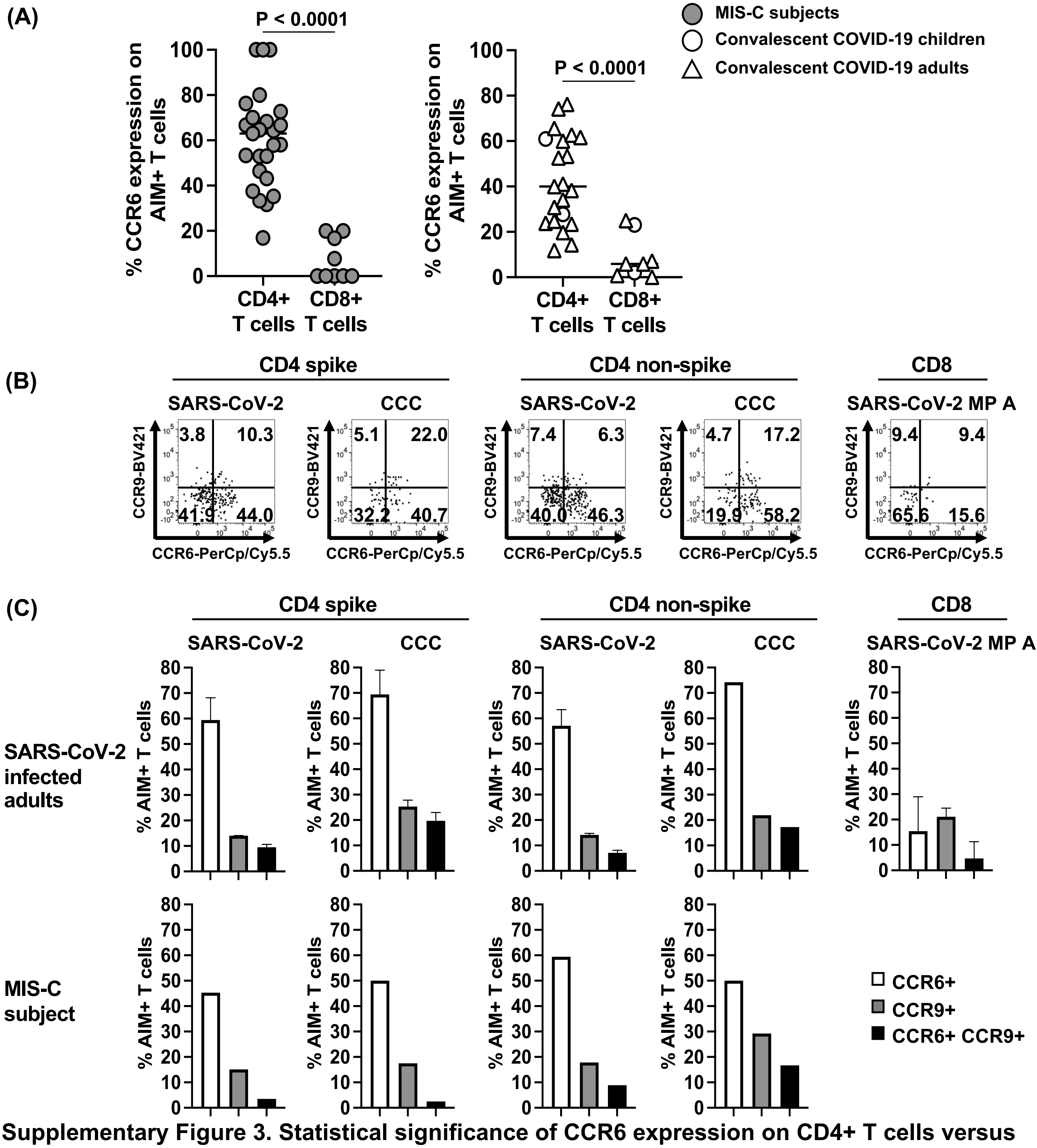

Supplementary Figure 3. Statistical significance of CCR6 expression on CD4+ T cells versus CD8+ T cells and co-expression of CCR9 on AIM+ CD4+ and CD8+ T cells. (A) CCR6 expressions on AIM+ CD4+ T cells and CD8+ T cells from MIS-C (left panels) and convalescent COVID-19 subjects (right panels). The expression of CCR6 on AIM+ CD4+ T cells was significantly higher than in CD8+ T cells from both MIS-C ($P < 0.0001$) and in convalescent COVID-19 subjects ($P < 0.0001$). Circles: pediatric subjects; triangles: adult subjects; gray symbols: MIS-C; white symbols: convalescent COVID-19 subjects. Mann-Whitney test was used to compare the CCR6 expression on AIM+ CD4+ and CD8+ T cells. A P value < 0.05 was considered to be significant. **(B)** Representative FACS plots showing the CCR6 and CCR9 expression on AIM+ CD4+ and CD8+ T cells in response to different CD4 and CD8 megapools from one convalescent COVID-19 subject. **(C)** Percentage of CCR6 single expression (white bars), CCR9 single expression (gray bars), and CCR6 and CCR9 co-expression (black bars) on antigen-specific AIM+ CD4+ and CD8+ T cells from two COVID-19 convalescent adults (upper panels) and one MIS-C subject (bottom panels).
